# C6 Ceramide (d18:1/6:0) as a Novel Treatment of Cutaneous T Cell Lymphoma

**DOI:** 10.3390/cancers13020270

**Published:** 2021-01-13

**Authors:** Raphael Wilhelm, Timon Eckes, Gergely Imre, Stefan Kippenberger, Markus Meissner, Dominique Thomas, Sandra Trautmann, Jean-Philippe Merlio, Edith Chevret, Roland Kaufmann, Josef Pfeilschifter, Alexander Koch, Manuel Jäger

**Affiliations:** 1 Department of General Pharmacology and Toxicology, Goethe University Hospital and Goethe University Frankfurt, 60590 Frankfurt am Main, Germany; eckes@em.uni-frankfurt.de (T.E.); imre@med.uni-frankfurt.de (G.I.); pfeilschifter@em.uni-frankfurt.de (J.P.); koch@med.uni-frankfurt.de (A.K.); 2 Department of Dermatology, Venerology and Allergology, Goethe University Hospital, 60590 Frankfurt am Main, Germany; kippenberger@em.uni-frankfurt.de (S.K.); markus.meissner@kgu.de (M.M.); roland.kaufmann@kgu.de (R.K.); manuel.jaeger@klinikum-karlsruhe.de (M.J.); 3 Department of Clinical Pharmacology, Goethe University Hospital and Goethe University Frankfurt, 60590 Frankfurt am Main, Germany; thomas@med.uni-frankfurt.de (D.T.); trautmann@med.uni-frankfurt.de (S.T.); 4 Cutaneous Lymphoma Oncogenesis Team, INSERM U1053 Bordeaux Research in Translational Oncology, Bordeaux University, 33076 Bordeaux, France; jp.merlio@u-bordeaux.fr (J.-P.M.); edith.chevret@u-bordeaux.fr (E.C.); 5 Hautklinik, Städtisches Klinikum Karlsruhe, Akademisches Lehrkrankenhaus der Universität Freiburg, 76133 Karlsruhe, Germany

**Keywords:** C6 ceramide, short-chain ceramide, cutaneous T cell lymphoma, skin

## Abstract

**Simple Summary:**

There is no curative treatment for mycosis fungoides and Sézary syndrome, which are the most frequent forms of cutaneous T cell lymphoma (CTCL). Short-chain ceramides like C6 Ceramide are known to induce cell death by both apoptosis and necrosis. Here, we demonstrate that C6 Ceramide strongly reduced cell viability and induced cell death in CTCL cell lines but not in HaCaT keratinocytes and primary human keratinocytes. C6 Ceramide was rapidly metabolized by both keratinocyte cell types but not by CTCL cells. These results provide the basis for further clinical trials with topical applicated C6 Ceramide against mycosis fungoides and Sézary syndrome.

**Abstract:**

Cutaneous T cell lymphomas (CTCLs) represent a heterogeneous group of T cell lymphomas that primarily affect the skin. The most frequent forms of CTCL are mycosis fungoides and Sézary syndrome. Both are characterized by frequent recurrence, developing chronic conditions and high mortality with a lack of a curative treatment. In this study, we evaluated the effect of short-chain, cell-permeable C6 Ceramide (C6Cer) on CTCL cell lines and keratinocytes. C6Cer significantly reduced cell viability of CTCL cell lines and induced cell death via apoptosis and necrosis. In contrast, primary human keratinocytes and HaCaT keratinocytes were less affected by C6Cer. Both keratinocyte cell lines showed higher expressions of ceramide catabolizing enzymes and HaCaT keratinocytes were able to metabolize C6Cer faster and more efficiently than CTCL cell lines, which might explain the observed protective effects. Along with other existing skin-directed therapies, C6Cer could be a novel well-tolerated drug for the topical treatment of CTCL.

## 1. Introduction

Cutaneous T cell lymphomas (CTCLs) are a group of extranodal non-Hodgkin T cell lymphomas that especially affect the skin and show an increasing standardized incidence of 1–9 per million people with regional variations [1,2]. The most frequent clinicopathological forms of CTCL are mycosis fungoides and Sézary syndrome. Their classification and diagnosis were described in the 2005 World Health Organization-European Organization for Research and Treatment of Cancer (WHO-EORTC) consensus classification [3] and the 2018 update of the WHO-EORTC [4].

Mycosis fungoides is marked by a rather indolent clinical behavior with a five-year disease-specific survival of 88%, whereas Sézary syndrome, a leukemic variant of CTCL, is characterized by aggressive clinical behavior with a five-year disease-specific survival of 36% [4,5]. Alibert Bazin type mycosis fungoides represents the most frequent variant of all primary cutaneous lymphomas and is dominated by lymphocytic infiltrates of epidermotropic small- to medium-sized T lymphocytes, most often with a T-helper phenotype [4,6]. These may lead to patches, plaques, tumors, or a combination thereof [7,8,9]. Patches manifest as erythematous lesions especially in sun-protected body areas and plaques are characterized by irregular erythematous or reddish-brown lesions of variable scaling. Tumors are able to ulcerate and show nodular or diffuse infiltrates involving the entire dermis [6]. According to the WHO-EORTC classification of CTCLs, folliculotropic mycosis fungoides, pagetoid reticulosis, and granulomatous slack skin are rare variants of mycosis fungoides with other clinicopathologic behaviors and outcomes [4]. Patients with Sézary syndrome typically suffer from pruritic erythroderma, generalized lymphadenopathy, and neoplastic T cells (Sézary cells) in skin, lymph nodes, and peripheral blood [4,10].

Early-stage (stages IA-IIA) mycosis fungoides patients receive primarily skin targeting therapy involving topical steroids, phototherapeutic treatments (narrow-band UV-B, Psoralen plus UV-A (PUVA)), and topical mechlorethamine [8,10,11,12]. Treatment options for patients with progressed mycosis fungoides at advanced stages (stages IIB-IVB) are combinations of skin-directed therapy and bexarotene, interferon-α and -γ, methotrexate, and extracorporeal photochemotherapy or photopheresis (ECP). Second-line therapy options include chemotherapy, total skin electron beam therapy (TSEBT), or allogeneic stem cell transplantation [8,11,12]. The remission rate, however, is much lower compared to early-stage mycosis fungoides [9]. Treatment with the antibodies mogamulizumab [13] and brentuximab [14] showed promising results in multicenter phase 3 studies. The severity of Sézary syndrome is also classified by different stages (I-IVB) and therapy options are similar to those of advanced stages of mycosis fungoides [8,12].

Despite the available treatments, CTCL is characterized by recurrence, chronic conditions, and high mortality. Furthermore, patients with CTCL suffer from adverse effects of the therapy, hospitalization, injections, and bad influence on psychiatric condition [15]. Therefore, it is necessary to research and develop new drugs and more efficient therapies for mycosis fungoides and Sézary syndrome, especially since there is no curative treatment yet [9].

Members of the class of sphingolipids are both structural components of the cell membrane with essential roles in barrier function and membrane fluidity and bioactive mediators that regulate basal cell processes [16,17,18,19]. The central molecules of the sphingolipid metabolism are the ceramides. Programmed cell death (apoptosis) can be triggered by the elevation of the concentration of specific ceramide species. Ceramide-activated Ser-Thr protein phosphatases (CAPPs) are downstream targets of ceramide and represent one way of ceramide-induced apoptosis. Activated CAPPs, such as protein phosphatase-2A (PP-2A), which is activated by both short- and long-chain ceramides [20,21], transmit the signal to several targets such as protein kinase B (AKT) [22], cyclin-dependent kinases (CDKs), and B-cell lymphoma-2 (bcl-2) [23]. The not naturally occurring short-chain ceramides (Cer) C2Cer and C6Cer induce apoptosis by activating caspase-3 and -7, poly (ADP-ribose) polymerase (PARP) cleavage, and cytochrome c release [24,25].

Much effort has been made to exploit the pro-apoptotic and tumor-suppressive effect of ceramide to treat different types of cancers by increasing the endogenous level of ceramide. The treatment with exogenous short-chain ceramides, such as C6Cer, turned out to be successful in many cancer studies [25,26,27,28,29,30]. C6Cer has been shown to induce cell death, both apoptosis and necrosis, in several cancer cell lines and in vivo [25,26,27,28,29,30]. In addition, short-chain ceramides were used in one phase 2 study to treat cutaneous breast cancer by topical application [31].

Mycosis fungoides is histopathologically characterized by epidermotropism of malignant T cells and is therefore predestined for topical treatments [6]. Since short-chain ceramides are highly skin permeable, they are suitable for topical applications and therefore promising to treat these forms of CTCLs [32]. Hence, we wanted to analyze and compare the effects of C6Cer on keratinocytes and two CTCL cell lines, MyLa (as a model for mycosis fungoides) and HuT78 (as a model for Sézary syndrome), to provide the basis for the employment of C6Cer as a treatment for cutaneous cancer and further in vivo studies.

## 2. Results

### 2.1. Effect of C6 Ceramide on Cell Viability of Keratinocytes and Cutaneous T Cell Lymphoma Cell Lines

**Figure 1:**
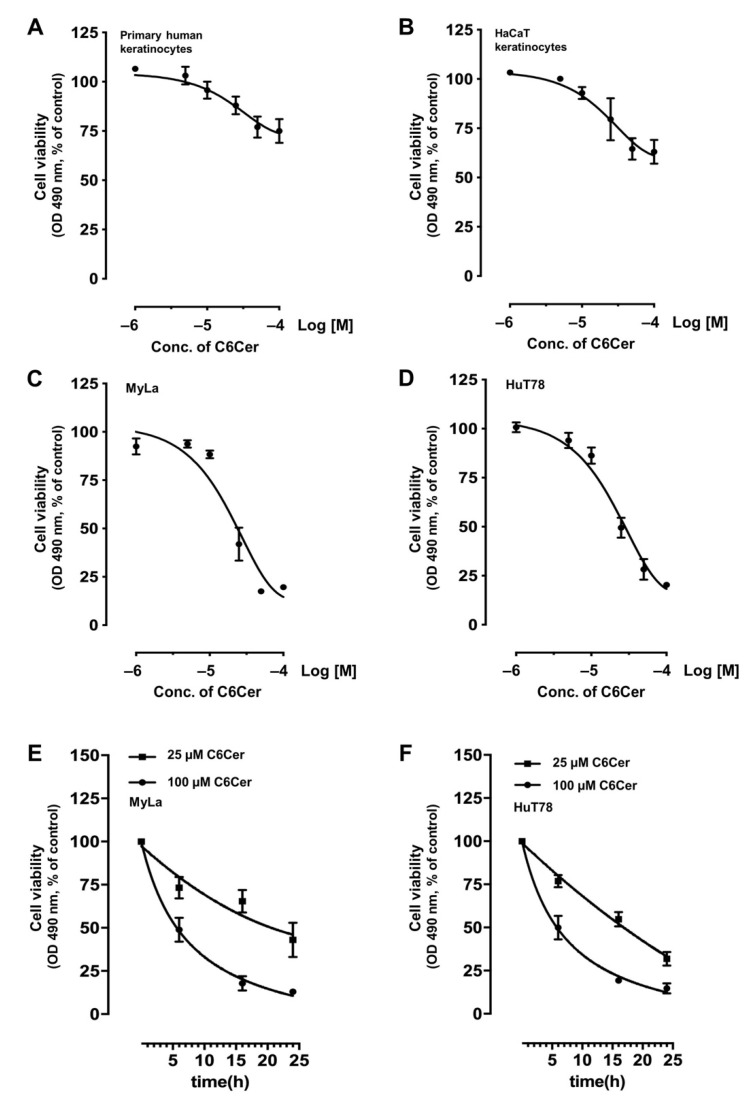
C6 ceramide decreases cell viability in keratinocytes and cutaneous T cell lymphoma cell lines. Dose-dependent effect of C6 ceramide (C6Cer) on cell viability of primary human keratinocytes (**A**) and HaCaT keratinocytes (**B**). Dose- and time-dependent effect of C6Cer on cutaneous T cell lymphoma cell lines MyLa (**C**,**E**) and HuT78 (**D**,**F**). The cells in A–D were treated with 1, 5, 10, 25, 50, and 100 µM C6Cer for 24 h. The cells in E and F were treated with 25 and 100 µM C6Cer for 6, 16, and 24 h. Cell viability was determined by MTS assay. All data are shown as means ± SEM (*n* = 3–7).

We investigated the effect of C6Cer on the cell viability of primary human keratinocytes (Figure 1A) and HaCaT cells (Figure 1B) with an MTS assay. If treated with increasing concentrations of C6Cer for 24 h (1–100 µM) primary human keratinocytes and HaCaT cells showed a dose-dependent decrease in cell viability. Treatment with 25 µM C6Cer for 24 h reduced cell viability of HaCaT cells by 37.5% and cell viability of primary keratinocytes by 28.8% compared to the respective control group. If treated with 100 µM for 24 h, a decrease in cell viability of 51.5% for HaCaT cells and 38.2% for primary keratinocytes was measured. Dose- and time-dependent effects of C6Cer treatment on cell viability of MyLa cells (Figure 1C,E) and HuT78 cells (Figure 1D,F) were also assessed by MTS assay. CTCL cell lines showed a dose-dependent decrease in cell viability (Figure 1C,D). When treated with 25 µM C6Cer for 24 h, a reduction of cell viability by 67.3% (MyLa) and 56.2% (HuT78) was measured compared to the control group. Incubation with 100 µM C6Cer for 24 h caused a reduction of cell viability by 91.4% (MyLa) and 89.9% (HuT78). Further, cells were treated with 25 µM and 100 µM for 6 h, 16 h, and 24 h whereby cell viability of both CTCL cell lines decreased to a similar extent. After treatment with 25 µM C6Cer for 6 h, 16 h, and 24 h, cell viability of MyLa cells dropped to 26.7%, 35.5%, and 57.0%, respectively. Treatment with 100 µM C6Cer for 6 h, 16 h, and 24 h reduced cell viability by 51.1%, 82.1%, and 87.0%. If HuT78 cells were treated with 25 µM for 6 h, 16 h, and 24 h, cell viability was reduced by 21.4%, 46.7%, and 63.9%, respectively, and, after the treatment with 100 µM for 6 h, 16 h, and 24 h by 52.4%, 77.1%, and 79.8% respectively (mean reduction of cell viability after 6 h, 16 h, and 24 h (%)).

### 2.2. Influence of C6 Ceramide on Necrosis, Apoptosis, and Autophagy in MyLa and HuT78 Cells

**Figure 2:**
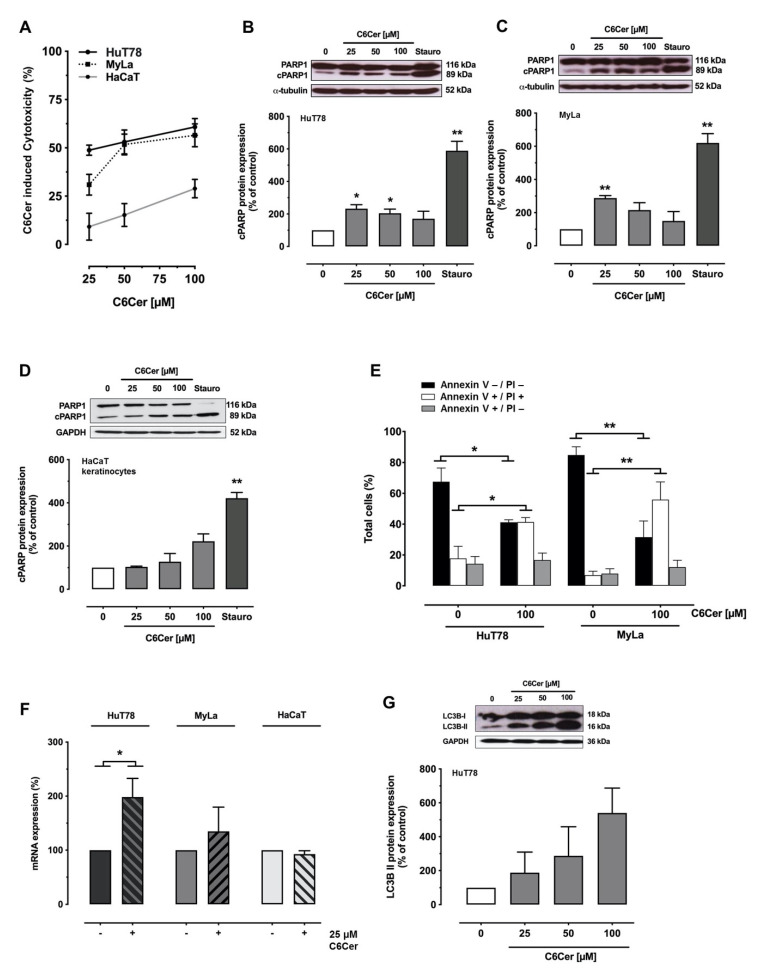
C6 ceramide treatment results in necrosis, apoptosis, and autophagy in cutaneous T cell lymphoma cell lines but not in HaCaT keratinocytes. (**A**): Cells were treated with C6 ceramide (C6Cer) for 24 h and lactate dehydrogenase was measured in the supernatant. (**B**–**D**): HuT78 (**B**), MyLa (**C**), and HaCaT (**D**) cells were treated with C6Cer, 1 µM staurosporine (STS), or vehicle for 24 h and poly (ADP-ribose) polymerase-1 (PARP1) cleavage was determined by Western Blot. (**E**): Cells were treated with C6Cer or vehicle for 24 h and cells analyzed by flow cytometry after Annexin V (ANXA5) and propidium iodide (PI) staining. ANXA5-, PI-: Healthy cells; ANXA5+, PI+: Late apoptotic or necrotic cells; ANXA5+, PI-: Early apoptotic cells. (**F**): Cells were treated with C6Cer or vehicle for 24 h. Microtubule-associated proteins 1A/1B light chain 3B (LC3B) mRNA expression was determined by TaqMan^®^ and normalized to the value of the vehicle-treated cells. (**G**): HuT78 cells were stimulated with C6Cer or vehicle for 24 h. LC3B-I and LC3B-II protein expressions were determined by Western Blot analysis. All data are shown as means ± SEM (A–F: *n* = 3–4; G: *n* = 2), * *p* < 0.05, ** *p* < 0.01. Abbreviations: Glycerinaldehyde-3-phosphate-Dehydrogenase (GAPDH).

Next, we investigated the details of the C6Cer triggered cytotoxic effect. Necrosis is a lytic cell death modality, accompanied by osmotic imbalances and early membrane ruptures. As an indicator for necrosis, we measured lactate dehydrogenase (LDH) release in the cell culture supernatant after treatment with 25, 50, and 100 µM C6Cer for 24 h (Figure 2A). In line with the results obtained by MTS assay, C6Cer treatment was cytotoxic for CTCL cells and HaCaT keratinocytes in a dose-dependent manner. After treatment with 25 µM C6Cer for 24 h, LDH assay results showed cytotoxicity of 30.9 ± 5.41% (MyLa), 48.8 ± 2.65% (HuT78), with 100 µM C6Cer for 24 h 56.5 ± 5.94% (MyLa) and 60.8 ± 4.35% (HuT78). In contrast, C6Cer treatment of HaCaT cells for 24 h led to cytotoxicity of only 9.16 ± 6.94% with 25 µM C6Cer and 28.9 ± 4.76% with 100 µM C6Cer treatment (mean ± SEM). As C6Cer led to low viability and high cytotoxicity in T cells but not in keratinocytes, we further investigated the dose-dependent effect of C6Cer on caspase-dependent poly (ADP-ribose) polymerase-1 (PARP1) cleavage as an indicator for apoptosis [33] in HuT78 (Figure 2B) and MyLa (Figure 2C) cells. Treatment with 25, 50, and 100 µM C6Cer for 24 h led to a significant increase of cleaved PARP1 (cPARP1) compared to the vehicle-treated control group. In contrast to the CTCL cells, HaCaT keratinocytes showed no significant increase in PARP1 cleavage (Figure 2D). 1 µM staurosporine (STS), an extensively used model compound for induction of apoptosis, served as a positive control for caspase-3 induced apoptosis. The whole blots showing all the bands and molecular weight markers are shown in Figure S1. To distinguish between apoptosis and necrosis in CTCL cells, we analyzed Annexin V (ANXA5) staining and propidium iodide (PI) incorporation in MyLa and HuT78 cells by flow cytometry after treatment with 100 µM C6Cer for 24 h. A significant decrease of the ANXA-, PI- cell population (healthy), and a significant increase of the ANXA5+ and PI+ (late apoptotic/necrotic) cell populations were measured. This reflects a reduction of healthy cells and an increase of necrotic or late apoptotic cells in both cell lines after the treatment with 100 µM C6Cer for 24 h (Figure 2E). There were no significant alterations in the early apoptotic cell population (ANXA+, PI-) after treatment with 100 µM C6Cer for 24 h. Furthermore, we looked for microtubule-associated proteins 1A/1B light chain 3B (LC3B) in T cells and HaCaT keratinocytes as a marker for autophagy flux and autophagosome biogenesis. T cells were treated with 25 µM C6Cer for 24 h and LC3B mRNA expression was measured by TaqMan^®^. A significant increase of LC3B mRNA expression was detected after treatment with 25 µM C6Cer for 24 h in HuT78 cells but not in MyLa cells and HaCaT keratinocytes (Figure 2F). Western Blot analysis of HuT78 cells treated with 25, 50, and 100 µM C6Cer for 24 h confirmed an increase of LC3B lipidation (Figure 2G). The whole blots are shown in Figure S2. Taken together, C6Cer treatment led to necrosis and apoptosis in MyLa and HuT78 cells and autophagy in HuT78 cells but not in HaCaT keratinocytes.

### 2.3. Metabolism of C6 Ceramide in HaCaT Keratinocytes and Cutaneous T Cell Lymphoma Cell Lines

**Figure 3:**
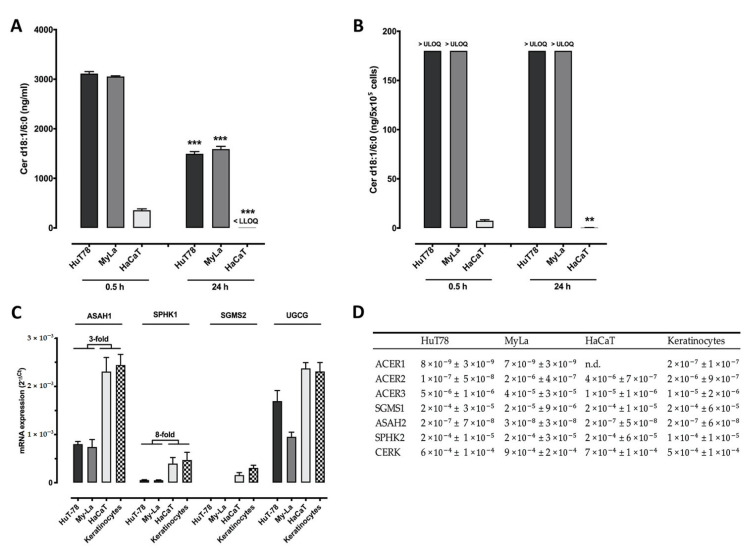
HaCaT keratinocytes catabolize C6 ceramide faster compared to cutaneous T cell lymphoma cell lines. (**A**,**B**): HaCaT cells were treated with 25 µM C6 ceramide (C6Cer), T cells with 12.5 µM C6Cer for 0.5 h and 24 h. The concentration of Cer d18:1/6:0 was measured by LC-MS/MS in the supernatant (**A**) and cell pellet (**B**). (**C**,**D**): Basal mRNA expression of acid ceramidase (ASAH) 1–2, sphingosine kinase (SPHK) 1–2, sphingomyelin synthase (SGMS) 1–2, Ceramide glucosyltransferase (UGCG), alkaline ceramidase (ACER) 1–3 and ceramide kinase (CERK) determined by TaqMan^®^ and shown as ΔCt values. Data are shown as means ± SEM (*n* = 3–4), ** *p* < 0.01, *** *p* < 0.0001. Abbreviations: Not detectable (n.d.); lower limit of quantification (LLOQ): 6 ng/mL; upper limit of quantification (ULOQ): 180 ng/pellet. *** in (**A**), ** in (**B**): significantly different compared to 0.5 h.

Since HaCaT keratinocytes proved to be more resistant against C6Cer compared to the CTCL cell line, we investigated the lipid metabolism and looked for differences in the efficacy of C6Cer degradation, which could protect against cell death. Therefore, HaCaT cells were incubated with 25 µM C6Cer for 0.5 h or 24 h and CTCL cell lines were treated with 12.5 µM C6Cer for the same time. C6Cer concentration was measured in the supernatant (Figure 3A) and the cell pellet (Figure 3B) by LC-MS/MS. There was a reduction of the C6Cer level in the supernatant after 24 h compared to 0.5 h of 51.9% (3113 ± 40.5 to 1495 ± 44.2 ng/mL) in HuT78 cells and 47.9% (3055 ± 13.3 to 1591 ± 54.0 ng/mL) in MyLa cells. In HaCaT cells, LC/MS-MS results showed a reduction of at least 98.3% (357 ± 37.3 to less than the lowest limit of quantification (LLOQ) of 6 ng/mL ± 0). The C6Cer level in the cell pellet of both CTCL cell lines after 0.5 h and 24 h were above the upper limit of quantification of 180 ng/sample. In HaCaT cells, LC/MS-MS results showed a reduction of 94.0% (7.5 ± 0.95 to 0.44 ± 0.05 ng/5 × 10⁵ cells) (mean 0.5 h ± SEM to mean 24 h ± SEM). To elucidate if enzymatic equipment is the reason for the differences in ceramide catabolism between keratinocytes and CTCL cell lines, we measured basal mRNA expression of involved enzymes (Figure 3C). Interestingly, mRNA expression of acid ceramidase (ASAH) 1 in primary human keratinocytes and HaCaT cells was threefold higher compared to T cells. Sphingosine kinase 1 (SphK1) mRNA was eightfold higher expressed in both keratinocyte cell lines compared to the CTCL cell lines. Sphingomyelin synthase (SGMS) 2 was barely expressed in T cells, whereas it was well expressed in both keratinocyte cell lines. However, absolute mRNA expressions of SPHK1 and SGMS2 were lower compared to ASAH1 expression. Ceramide glucosyltransferase (UGCG) was highly expressed in all cell lines except for MyLa cells. The mRNA expression of ASAH2, SPHK2, SGMS1, ceramide kinase (CERK), and alkaline ceramidase (ACER) 1–3 showed low expressions in all cell lines or no considerable distinctions between keratinocytes and CTCL cell lines (Figure 3D).

### 2.4. The Role of Acid Ceramidase 1 in C6 Ceramide Metabolism

**Figure 4:**
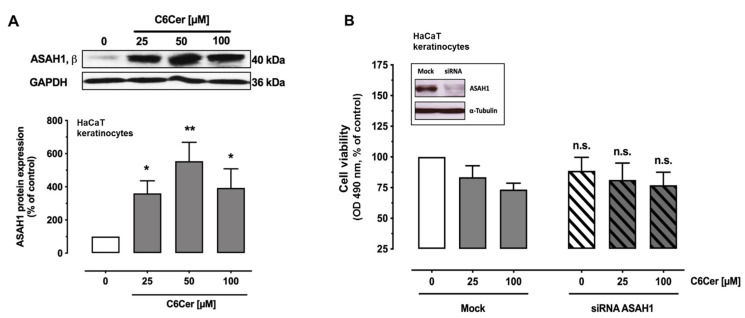
The role of acid ceramidase 1 in C6 ceramide metabolism. (**A**): HaCaT cells were treated with C6 ceramide (C6Cer) or vehicle for 24 h and protein level of ASAH1 β chain was determined by Western Blot. (**B**): Cells were treated with ASAH1- or mock small interfering RNA (siRNA) for 24 h. After incubation cells were treated with C6Cer or vehicle for 24 h. Then cell viability was measured by MTS assay and normalized to the value of the vehicle-treated mock group. The success of knockdown was proven by Western Blot analysis. Data are shown as means ± SEM (*n* = 3–4), * *p* < 0.05, ** *p* < 0.01. * and ** in A: Significantly different compared to the control group; n.s. in B: Not significantly different compared to the related mock-treated group.

As mass spectrometry results showed that the synthetical C6Cer was metabolized by the cells, we wanted to know whether the reason for the resistance of keratinocytes to C6Cer-induced cell death is due to the ceramide degrading enzyme ASAH1. HaCaT cells were treated with C6Cer for 24 h and ASAH 1 protein expression was measured. Interestingly, the protein expression of ASAH1 was significantly upregulated compared to the vehicle group (Figure 4A). The whole blots are shown in Figure S3. Because ASAH1 could play a critical role in protecting keratinocytes from C6Cer-induced cell death, we knocked down the enzyme by siRNA. Cells were incubated with ASAH1 siRNA and mock siRNA for 24 h before they were stimulated with C6Cer for 24 h. Knockdown was validated by Western Blot analysis. The whole blots are shown in Figure S4. There were no significant differences in cell viability between mock and ASAH1 siRNA treated groups (Figure 4B). To verify the data by a different approach, HaCaT cells were stimulated with 25 µM C6Cer, 15 µM of the highly potent ASAH1 inhibitor HCFU (1-hexylcarbamoyl-5-fluorouracil or Carmofur) or both. After an incubation time of 24 h, cell viability was determined by MTS assay. There was, again, no significant decrease in cell viability observed when treated with the ASAH1 inhibitor plus C6Cer (co: 100 ± 0; 25 µM C6Cer = 91.3 ± 6.65%; 15 µM HCFU = 114 ± 6.72%; 25 µM C6Cer and 15 µM HCFU = 101 ± 5.79% (mean ± SEM)).

## 3. Discussion

To date, there is no curative treatment of mycosis fungoides or Sézary syndrome [9]. As CTCLs are chronic diseases with frequent recurrence, patients must undergo treatment for their entire remaining life. Therefore, it is important to find new efficient treatments.

In our study, we demonstrated the potential of short-chain C6Cer to treat mycosis fungoides and Sézary syndrome. C6Cer treatment led to apoptosis and necrosis in MyLa and HuT78 cell lines. HuT78 cells showed a dose-dependent increase of autophagic flux in presence of C6Cer, which indicates vigorous cell stress and damage induced by C6Cer [34,35]. The results obtained from LDH assay and flow cytometry analysis indicate that the major cause for cell death is due to necrosis (Figure 2). In our experiments a significant increase in apoptotic PARP cleavage was observed, and, therefore, it cannot be excluded that the detected necrosis represents a late phase of apoptotic cell death. The predominance of necrosis was already shown in C6Cer treated Jurkat cells, a T cell leukemia cell line [26].

Further, we showed that HaCaT cells and primary human keratinocytes were less sensitive to C6Cer compared to CTCL cells. No significant increase in PARP cleavage or autophagic flux was observed in HaCaT keratinocytes. In this context, we were able to find differences in the efficiency of catabolizing C6Cer between HaCaT keratinocytes and CTCL cell lines. For lipid analysis, we treated HaCaT keratinocytes with 25 µM C6Cer and CTCL cell lines only with 12.5 µM to save most cells from dying. Already after 30 min, HaCaT cells catabolized most of C6Cer and after 24 h the concentrations in supernatant and pellet were dramatically decreased. Although CTCL cell lines were stimulated with less C6Cer, the lipid concentrations measured in samples from CTCL cell lines were always higher compared to the values measured in samples from HaCaT cells. Hence, HaCaT keratinocytes might be less sensitive to C6Cer-induced cell death due to their ability to catabolize it more efficiently than CTCL cell lines. Although ASAH1 mRNA expression was markedly upregulated in HaCaT keratinocytes after treatment with C6Cer, there was no impact on C6Cer-induced cell death by ASAH1 knockdown or pharmacological inhibition. However, other enzymes could compensate for the loss of ASAH1 and it is likely that several enzymes are involved in the metabolization of short-chain ceramides such as sphingomyelin synthases and ceramide glucosyltransferase [36].

Short-chain C6Cer is able to penetrate cells and skin and accumulates in cell membranes as well as in intracellular membranes [32,37]. Therefore, a topical C6Cer treatment of CTCL is technically possible. Topical application of short-chain ceramides as a treatment for cancer was already tested for C2Cer and C6Cer in phase 2 study of cutaneous breast cancer with twenty-five patients. Pruritis, rash, and dry skin were the most frequent side effects of short-chain ceramides, but no grade 3 or 4 toxicity was reported [31]. This is congruent with our in vitro data as HaCaT cells and primary human keratinocytes showed less cytotoxicity after C6Cer treatment compared to malignant T cells. Due to limited efficacy, the results of the cutaneous breast cancer trial were not promising enough to warrant further studies [31]. Because of high costs, they restricted their concentration to only a 1% mixture of C2Cer- and C6Cer [31] and transdermal drug application is limited by permeation of the stratum corneum, higher concentrations, and skin permeation enhancers, which induce a reversible decrease of the barrier resistance [38] and could be used to enhance the antitumoral effect of C6Cer in further in vivo trials.

Present topical therapy options consist above all of glucocorticoid and mechlorethamine [8,9]. Adverse effects of mechlorethamine include immediate hypersensitivity reactions (allergy), erythema, hyperpigmentation, and pruritis. Secondary malignancies like cutaneous melanoma, nonmelanoma skin cancer, and primary malignancies were also reported [39,40]. Glucocorticoids may trigger tachyphylaxis [41] and, as CTCL patients are treated for a long time, their therapeutic potential is therefore limited. Since glucocorticoids are an essential component in CTCL therapy [8], patients suffer from their local and systemic adverse effects. The most often adverse effects of topical steroids include cutaneous atrophy, local irritation, and skin depigmentation. Delayed wound healing and exacerbations of skin infections after treatment with glucocorticoids were also reported [42,43,44].

Therefore, it is necessary to find more efficient therapies with fewer adverse effects. From our data, we would suggest topical treatment with C6Cer as a potential topical treatment of mycosis fungoides and Sézary syndrome and as an alternative for patients with side effects or contraindications of other topical treatment options. Luckily adverse effects of C6Cer treatment were already investigated and seemed to be rather mild [31].

In conclusion, we could show that C6Cer significantly reduced cell viability and induced cell death in CTCL cell lines. Therefore, C6Cer offers a possible novel treatment of CTCL, which should be investigated in vivo. Furthermore, C6Cer treatment could be also studied for other skin associated diseases like dermal cancer and chronic inflammatory disorders with lymphocytic infiltrates.

As C6Cer treated cells were stimulated under aqueous cell culture conditions in this study, the results are limited to this in vitro model and the effect of topically applicated C6Cer on CTCLs has to be further investigated in vivo. In particular, it is questionable which concentrations can be applied for topical treatment of CTCLs. Furthermore, we focused on the comparison of CTCL cells with keratinocytes after the C6Cer treatment to elucidate the basis for clinical trials without affecting healthy skin. Therefore, this study is limited by the characterization of the detailed mechanism of C6Cer induced apoptosis in CTCL cell lines. We focused especially on the extrinsic pathway of apoptosis to compare the effect of C6Cer. Thus, we did not show the intrinsic pathway, alterations in mitochondria or an increase of reactive oxygen species, which has already been shown in previous studies [24,25,26,45].

## 4. Materials and Methods

### 4.1. Cell Culture

Sézary syndrome cell line HuT78 was a gift from Michael U. Martin (Justus-Liebig-Universität Gießen, Gießen, Germany), mycosis fungoides cell line MyLa was a gift from Jean-Philippe Merilo and Edith Chevret (University of Bordeaux, Bordeaux, France), HaCaT cells were a gift from Norbert Fusenig (German Cancer Research Institute, Heidelberg, Germany). HuT78, and MyLa cells were grown in RPMI 1640 medium [-] L-Glutamine with 1% penicillin/streptomycin, 1% L-Glutamine (all Gibco/Thermo Fisher, Waltham, MA, USA), 10% fetal calf serum (FCS) (Sigma-Aldrich, St. Louis, MO, USA). HaCaT cells in DMEM, high glucose, GlutaMAX^TM^ (Gibco/Thermo Fisher, Waltham, MA, USA) with 1% penicillin/streptomycin, 10% FCS. Normal primary human keratinocytes were isolated from infantile foreskins of donors and seeded in DermaLife^®^ K cell medium (CellSystems, Troisdorf, Germany). All cells were incubated at 37 °C in a humidified 5% CO_2_ incubator.

### 4.2. Chemicals

C6Cer (d18:1/6:0) (N-hexanoyl-D-erythro-sphingosine) (Avanti Polar Lipids, Alabaster, AL, USA and Cayman Chemical, Ann Arbor, MI, USA) was dissolved in DMSO to obtain 1000× stock solutions from 1–100 mM and stored immediately at −20 °C. Carmofur was obtained from Cayman Chemicals (Ann Arbor, MI, USA), staurosporine from Merck (Darmstadt, Germany) both dissolved in DMSO stored at −20 °C.

### 4.3. Cell Viability

To measure cell viability, we used the CellTiter 96^®^ AQ_ueous_ One Solution Cell Proliferation kit (MTS) (Promega, Madison, WI, USA). 3 × 10^4^ cells (Figure 1) and 10^5^ HaCaT cells per well (Figure 4B) were seeded in 96-well plates in 100 µL volume. 10 µL MTS was added and incubated at 37 °C for 30 min or 1 h. The absorbance at 490 nm was recorded using the microplate reader SpectraMax M5 (Molecular Devices, San Jose, CA, USA).

### 4.4. Cytotoxicity

Cytotoxicity was determined by Cytotoxicity Detection Kit (LDH) (Roche/Sigma Aldrich, St. Louis, MO, USA). Cytotoxicity (%) was determined as described in the assay protocol. Triton^TM^ X-10 (Sigmar-Aldrich, St. Louis, MO, USA) was used for positive control.

### 4.5. Western Blot Analysis

Immunoblotting was performed as described previously [46]. The following antibodies were used: rabbit anti-LC3B (NB100-2220, novus biologicals, Centennial, CO, USA); rabbit anti-ASAH1 antibody (HPA005468, Sigma-Aldrich, St. Louis, MO, USA); rabbit anti-PARP and anti-cPARP antibodies (#9542, Cell Signaling Tech., Danvers, MA, USA); goat anti-GAPDH (sc-20357, Santa Cruz Biotechnology, Dallas, TX, USA); mouse anti-alpha-Tubulin (#3873, Cell Signaling Tech., Danvers, MA, USA).

### 4.6. Flow Cytometry after Annexin V and Propidium Iodide Staining

To measure the fraction of healthy, early apoptotic, and late apoptotic/necrotic cells, we used an apoptosis detection kit (ALX-850-253-KI02, Enzo Life Sciences, Inc., Farmingdale, NY, USA). 3 × 10^5^ cells were seeded in 1 mL per well (6 well plate). After the respective treatment, 300 µL cell suspension was collected in a glass tube (Becton Dickinson, Franklin Lakes, NJ, USA). 3 µL of Annexin V, 3 µL Propidium Iodide and 0.75 µL 1 M CaCl_2_ dissolved in H_2_O were added and incubated by the exclusion of light for 15 min. The stained samples were measured by flow cytometry. Following channels were used for flow cytometry: FITC-FL1 channel (488 nm blue laser/530 nm bandpass filter), PI-FL2 channel (488 nm blue laser/585 nm bandpass filter). BD FACSCanto^TM^ II, Becton Dickinson, Franklin Lakes, NJ, USA).

### 4.7. Reverse Transcriptase Polymerase Chain Reaction (RT-PCR) and Real-Time Quantitative PCR (qPCR)

1.2 µg of total RNA was isolated with TRIZOL^TM^ reagent (Sigma-Aldrich, St. Louis, MO, USA) following the manufacturer’s protocol. Reverse transcriptase-polymerase chain reaction (RT-PCR) was performed with cDNA RevertAid^TM^ first-strand cDNA synthesis kit (Thermo Fisher, Waltham, MA, USA) by utilizing a random hexamer primer (Thermo Fisher, Waltham, MA, USA) for amplification to synthesize complementary DNA (cDNA). Real-time PCR (TaqMan^®^) was performed using the Applied Biosystems^®^ 7500 Fast Real-Time PCR System. The TaqMan^®^ system, all probes, primers, the reporter dyes 6-FAM and VIC, and the software were from Life Technologies (Thermo Fisher, Waltham, MA, USA). Following TaqMan^®^ assays were used: ASAH1, Hs01001661_m1; ASAH2, Hs01015663_m1; SPHK1, Hs00184211_m1; SPHK2, Hs00219999_m1; ACER1, Hs00370322_m1; ACER2, Hs04996319_g1; ACER3, Hs00924388_m1; CERK, Hs00368483_m1, SGMS1, Hs00380453_m1, SGMS2, Hs00983630_m1; UGCG, Hs00916612_m1; MAP1LC3B, Hs00797944_s1; Eukaryotic 18S rRNA, Hs9999990_s1 (all Thermo Fisher, Waltham, MA, USA).

### 4.8. Liquid Chromatography-Tandem Mass Spectrometry (LC-MS/MS)

HaCaT cells were grown in 60 × 15 mm cell culture dishes. HuT78 and MyLa cells were grown in 6 well culture plates. After the respective treatment for the indicated time, supernatants of HaCaT cells were taken and cell pellets were washed once with cold Dulbecco’s phosphate-buffered saline (DPBS) (Thermofisher, Waltham, MA, USA) after trypsinization and stored at −80 °C. T cells were centrifuged before supernatants were taken and cell pellets were washed once with DPBS and stored at −80 °C. Before storage, the amounts of healthy cells and total cells were determined with a TC20^TM^ automated cell counter (Bio-Rad Laboratories, Inc., Hercules, CA, USA). Ceramide analyses were done using liquid chromatography-electrospray ionization tandem mass spectrometry (LC-ESI-MS/MS) as described elsewhere [47].

In brief, cell samples were resuspended in 200 µL extraction buffer (citric acid 30 mM, disodium hydrogen phosphate 40 mM) and mixed with 20 µL internal standard solution (2 µg/mL, Cer d18:1/8:0 and GlcCer d18:1/8:0 in chloroform:methanol (2:1, *v/v*)). The samples were then extracted once with 600 µL methanol:chloroform:HCl (15:83:2, *v/v/v*). The lower organic phase was transferred and evaporated at 45 °C under a gentle stream of nitrogen and reconstituted in 200 µL tetrahydrofuran:water (9:1, *v/v*) containing 0.2% formic acid and 10 mM ammonium formate. For the supernatant, a 50 µL sample was mixed with 150 µL extraction buffer and extracted as described above. For calibration standards and quality control samples preparation, 20 µL of the corresponding working solutions were processed as stated instead of sample. The quantification of all analytes was performed using a hybrid triple quadrupole-ion trap mass spectrometer QTRAP 5500 (Sciex, Darmstadt, Germany) equipped with a Turbo-V-source operating in positive ESI mode. Ceramides were separated using an Agilent 1290 HPLC system equipped with a Zorbax C18 Eclipse Plus UHPLC column (2.1 × 50 mm, 1.8 μm, Agilent technologies, Waldbronn, Germany). Quality control samples of three different concentration levels (low, middle, high) were run at the beginning and end of each run. Samples were processed using Analyst software 1.6 and the obtained concentrations were evaluated using MultiQuant Software 3.0 (both Sciex, Toronto, ON, Canada) using the internal standard method (isotope-dilution mass spectrometry).

The calibration curve was calculated by quadratic regression with 1/x^2^ weighting. Variations in the accuracy of the calibration standards were lower than 15% over the range of calibration, except for the lower limit of quantification (LLOQ), where a limit of 20% was accepted. All measured data were collected in one LC-MS/MS measurement.

### 4.9. Knockdown Studies with Small Interfering RNA

Cells were transfected with small interfering RNA (siRNA) of ASAH1 and nontargeting siRNA as a negative control. (Hs.527412 (ASAH1) Assay ID 119213 Silencer^®^ and Silencer^TM^ Negative Control No.1 siRNA, AM461 (all Thermo Fisher, Waltham, MA, USA)). The transfection tool was Lipofectamine^TM^ 3000 Thermo Fisher, Waltham, MA, USA). Instructions of the manufacturer were followed. The success of the knockdown was verified by Western Blot analysis.

### 4.10. Statistics

Statistical significance was determined by an unpaired *t*-test. All statistics were evaluated with the program GraphPad^®^ Prism (Version 8.4.2 (464), GraphPad Software, San Diego, CA, USA).

## 5. Conclusions

In this study, we could show that C6Cer significantly reduced cell viability and induced cell death in CTCL cell lines but not in HaCaT keratinocytes and primary human keratinocytes. HaCaT keratinocytes seemed to be protected due to their ability to metabolize proapoptotic C6Cer more efficiently. Therefore, the application of C6Cer offers a possible novel topical treatment of CTCL.

## Data Availability

Data is contained within the article or supplementary material.

## Supplementary Materials

Supplementary Materials can be found at https://www.mdpi.com/2072-6694/13/2/270/s1, Figure S1: Western Blots of Figure 2B–D, Figure S2: Western Blot of Figure 2G, Figure S3: Western Blots of Figure 4A, Figure S4: Western Blots of Figure 4B.

## Abbreviations

C6Cer	C6 Ceramide (d18:1/6:0, N-hexanoyl-D-erythro-sphingosine)
CTCL	Cutaneous T cell lymphoma
ASAH	Acid ceramidase
LC-MS/MS	Liquid chromatography-tandem mass spectrometry
LC3B	Microtubule-associated proteins 1A/1B light chain
LDH	Lactate dehydrogenase
PARP	Poly (ADP-ribose) polymerase
