# Supplementary material for: C6 Ceramide (d18:1/6:0) as a Novel Treatment of Cutaneous T Cell Lymphoma
Source: Cancers (Basel). 2021 Jan 13;13(2):270. doi: 10.3390/cancers13020270 (PMC7828274; doi:10.3390/cancers13020270)
Supplement: Supplementary file 1 [file cancers-13-00270-s001.pdf]

Supplementary Materials

C6 Ceramide (d18:1/6:0) as a Novel Treatment of Cutaneous T Cell Lymphoma

Raphael Wilhelm, Timon Eckes, Gergely Imre, Stefan Kippenberger, Markus Meissner, Dominique Thomas, Sandra Trautmann, Jean-Philippe Merlio, Edith Chevret, Roland Kaufmann, Josef Pfeilschifter, Alexander Koch and Manuel Jäger

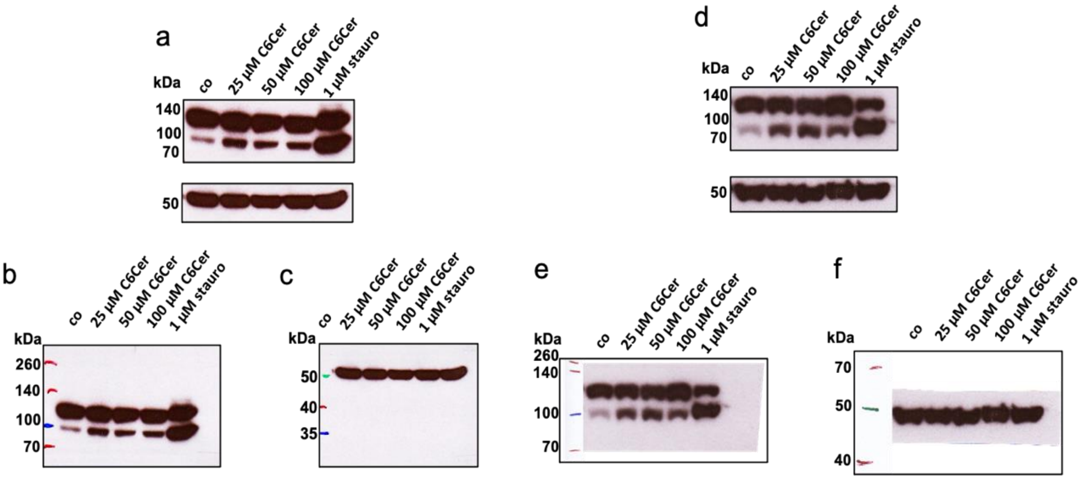

Figure S1. MyLa (**a–c**) and HuT78 (**d–f**) cells were treated with 25, 50, 100 µM C6Cer, 1 µM staurosporine (STS) or vehicle for 24 h and poly (ADP-ribose) polymerase-1 (PARP1) cleavage was determined by Western Blot. Rabbit anti-PARP and anti-cPARP antibodies (#9542, Cell Signaling Tech., Danvers, MA, USA) were used in a,b (MyLa cells) and d,e (HuT78 cells). Mouse anti-alpha-Tubulin (#3873, Cell Signaling Tech., Danvers, MA, USA) was used in a,c (MyLa cells) and d,f (HuT78 cells).

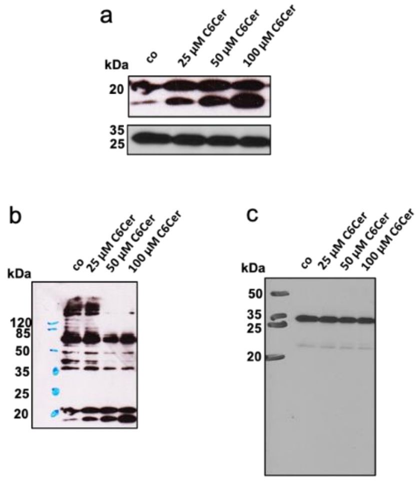

Figure S2. HuT78 cells were stimulated with 25, 50, 100 µM C6Cer or vehicle for 24 h. LC3B-I and LC3B-II protein expressions were determined by Western Blot analysis. Rabbit anti-LC3B (NB100-2220, novus biologicals, Centennial, CO, USA) was used in (**a,b**). Goat anti-GAPDH (sc-20357, Santa Cruz Biotechnology, Dallas, TX, USA) was used in (**a,c**).

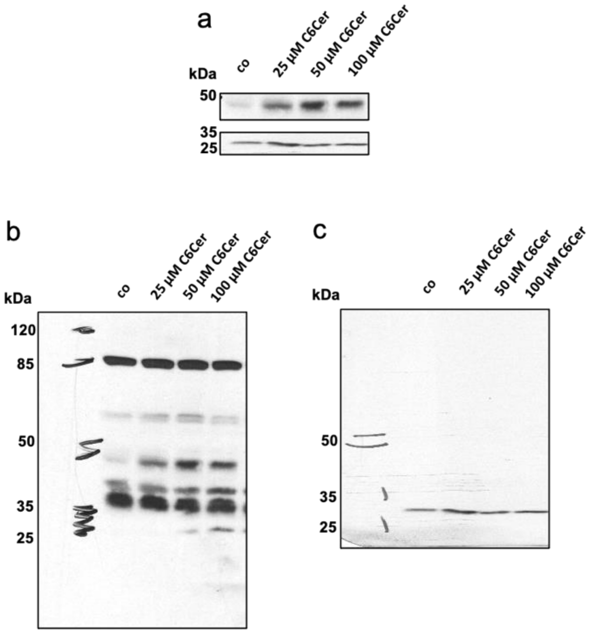

Figure S3. HaCaT cells were treated with C6Cer or vehicle for 24 h and protein level of ASAH1 β chain was determined by Western Blot. Rabbit anti-ASAH1 antibody (HPA005468, Sigma-Aldrich, St. Louise, MO, USA) was used in (**a,b**). Goat anti-GAPDH (sc-20357, Santa Cruz Biotechnology, Dallas, TX, USA) was used in (**a,c**).

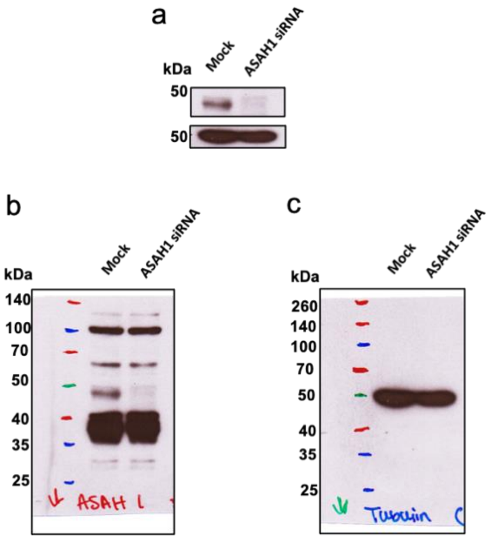

Figure S4. HaCaT cells were treated with ASAH1- or mock small interfering RNA (siRNA) for 24 h. Rabbit anti-ASAH1 antibody (HPA005468, Sigma-Aldrich, St. Louise, MO, USA) was used in (**a,b**). Goat anti-GAPDH (sc-20357, Santa Cruz Biotechnology, Dallas, TX, USA) was used in (**a,c**).

© 2021 by the authors. Submitted for possible open access publication under the terms and conditions of the Creative Commons Attribution (CC BY) license (http://creativecommons.org/licenses/by/4.0/).
